# Prevalence and Predictors of Sleep Disturbance, Anxiety and Depression among Patients with Chronic Respiratory Diseases

**DOI:** 10.3390/ijerph191912819

**Published:** 2022-10-06

**Authors:** Yousef S. Aldabayan, Jaber S. Alqahtani, Ahmed M. Al Rajeh, Amal Ismael Abdelhafez, Rayan A. Siraj, Vidhya Thirunavukkarasu, Abdulelah M. Aldhahir

**Affiliations:** 1 Department of Respiratory Care, King Faisal University, Al Ahsa 31982, Saudi Arabia; 2 Department of Respiratory Care, Prince Sultan Military College of Health Sciences, Dammam 34313, Saudi Arabia; 3 Department of Nursing, King Faisal University, Al Ahsa 31982, Saudi Arabia; 4 Department of Critical Care & Emergency Nursing, Assiut University, Asyut 71717, Egypt; 5 Respiratory Therapy Department, Jazan University, Jazan 45141, Saudi Arabia

**Keywords:** sleep quality, anxiety, depression, respiratory diseases

## Abstract

Background: Poor sleep quality, depression, and anxiety are common comorbidities among individuals with chronic respiratory diseases (CRDs). However, there has been no work to estimate their prevalence and assess their associations among the CRDs population in Saudi Arabia. Methods: A cross-sectional study was conducted in primary healthcare centers and included a total of 390 patients. Structured self-administered questionnaires were completed that included the Sleep Quality Pittsburgh Sleep Quality Index (PSQI) and Hospital Anxiety and Depression Score (HADS). Multiple linear regression analyses were performed to assess the associations between patients’ characteristics and sleep disturbance, anxiety and depression. Results: Poor sleep quality, depression, and anxiety affect 75%, 49.2%, and 36.4% of the study participants, respectively. The PSQI was significantly correlated with anxiety (r = 0.30) and depression (r = 0.16). Furthermore, a significant correlation was found between anxiety and depression (r = 0.44). The predictors of poor sleep quality were age, gender, and family history of CRDs, education level and anxiety and these variables accounted for 0.19% of the variance in PSQI. Variables that independently predicted an increased level of depression were age, gender, marital status, family history of CRDs, diagnosis, previous hospital admission, the presence of comorbidities, dyspnea last month and anxiety. On the other hand, the variables that independently predicted an increased level of anxiety were age, BMI, family history of CRDs, previous hospital admission, the presence of comorbidities, dyspnea last month and depression. Conclusion: Healthcare providers managing patients with CRDs should be alert to the high prevalence of poor sleep quality, depression, and anxiety. Appropriate interventions to reduce the prevalence should be developed and timely applied.

## 1. Introduction

Chronic respiratory diseases (CRDs) are among the most common non-communicable diseases worldwide, largely due to the ubiquity of noxious environmental, occupational, and smoking exposures [1]. Some of the most common CRDs are chronic obstructive pulmonary disease (COPD), asthma, occupational lung diseases, and pulmonary hypertension [2]. CRDs represent a public health challenge in both developed and developing countries and are associated with impairment in daily life activities, social functioning, psychological, and increased the risk of morbidity and mortality [3].

Worldwide, it was noted that there is a noticeable increase in the prevalence of CRDs in different populations, with a prediction of increasing the global burden of CRDs in the future [4]. Moreover, the adverse outcomes of the burden of CRDs on the well-being and quality of life of affected individuals was pointed out from several aspects [5]. Therefore, the government of Saudi Arabia is determined to enhance the quality of preventive and therapeutic healthcare services as a part of its Vision 2030 [6].

Patients with CRD experience severe sleep disturbance, anxiety, and depression due to frequent hospitalization and the breathlessness they often experience. The adverse effects on breathing include disturbances in respiratory control, respiratory muscle function, and lung mechanics [7]. Comorbid sleep disorders portend worse sleep quality, diminished quality of life, and numerous other adverse consequences. Although there is a lack of understanding of the etiologies, impacts, and therapies of sleep disorders, specifically in patients with CRDs [8,9,10], it seems likely that the resultant physiological effects are due to hypoventilation with associated hypoxemia and hypercapnia. Whereas these symptoms may be mild and clinically insignificant in normal subjects, those with CRDs (e.g., COPD) may experience serious physiological changes during sleep, leading to clinically significant disturbances in gas exchange [11].

Patients with CRD have a higher prevalence of anxiety and depression than the general population, leading to worse clinical outcomes. However, the psychological problems are often left undiagnosed and not managed [12].

When the patient experiences frequent episodes of breathlessness, it creates an anxious and threatening life situation, lack of assurance and physiologic triggering, and a sense of helplessness which tends to have pervasive thinking about their life, sequentially leading to depression. Further, psychological disorders such as depression and anxiety are interlinked and common in patients with CRDs, and they play an indispensable role in increasing the risk of hospitalization, poorer outcomes, increased mortality, poorer quality of life, and social functioning [13].

Depressed patients are more likely to have episodes of worsening respiratory difficulties symptoms than non-depressed patients, and even mild co-morbid depression is associated with a twofold higher use of emergency hospital resources [14]. Depression and anxiety are commonly co-morbid in patients with CRDs. For example, the result of the study by Husain MO and Chaudhry (2021) highlighted that depressed COPD patients who had reported more perceived stressful life events and a lack of social support from family, friends, and colleagues, leading to feelings of sadness, anger, and in some cases social isolation, were more likely to report depression and poor health-related quality of life [15]. To date, some studies have been conducted to report the prevalence of poor sleep quality, depression, and anxiety with a different focus, including medical students and children but not among patients with CRDs in Saudi Arabia. Thus, the objective of this study is to identify the prevalence of sleep disturbance, anxiety, and depression among patients with CRDs and to determine the associations between and predictors of sleep quality, anxiety, and depression.

## 2. Materials and Methods

### 2.1. Study Design and Setting

A cross-sectional study was conducted in primary healthcare centers with respiratory and smoking clinics in Alhufof city, Al-Ahsa, which is located in the eastern province of Saudi Arabia.

### 2.2. Power Calculation

A total of 390 patients diagnosed with CRDs (asthma and chronic obstructive pulmonary disease) were enrolled from October 2021 through March 2022. The inclusion criteria were aged > 18 years, stable health status, able to comprehend and verbalize, no mental disorders, and willingness to participate. Patients were excluded if they were in acute or exacerbation condition, not confirmed diagnostically. The sample size was calculated using open source epidemiologic statistics from a public health website (www.OpenEpi.com, Open Source Epidemiologic Statistics for Public Health. Available onlinehttps://www.openepi.com/SampleSize/SSPropor.htm accessed on 5 September 2021).

The total population was 316,841 (planning department, 2022), with a 95% confidence level and 80% power, and the total sample size was 386. This number was then rounded up to 390. Therefore, the power sample size was based on the prevalence outcomes.

### 2.3. Data Collection Questionnaire and Procedure

A structured self-administered questionnaire was used to collect the data. It was composed of a section about the patients’ characteristics and two standardized tools. The characteristics of the patients included age, gender, marital status, education level, BMI, family history of respiratory disease, smoking history, previous hospital admission due to exacerbation, and comorbidities. The first tool was the Sleep Quality Pittsburgh Sleep Quality Index (PSQI) developed by Buysse et al., which was used to assess subjective sleep disturbances over a one-month time interval [16]. It is a standardized questionnaire, and its reliability and validity were measured in previous study [17,18]. Here, we used the Arabic version [19]. The PSQI is a 19-item self-rating scale designed to measure the perceived quality of sleep during the preceding month. It provides seven component scores comprising (1) subjective sleep quality, (2) sleep latency, (3) sleep duration, (4) habitual sleep efficiency, (5) sleep disturbances, (6) use of sleeping medication, and (7) daytime dysfunction. Each item is weighted on a 0–3 interval scale, with a higher score indicating worse sleep quality. Global scores range from 0 to 21, with scores greater than five indicating poor sleep quality and those ≤ 5 indicating good sleep quality [16].

The second tool was the Hospital Anxiety and Depression Score (HADS), which is a self-report tool that is frequently used in non-psychiatric settings to detect the two most frequent expressions of distress: anxious and depressive states [20]. The HADS consists of seven questions for anxiety and seven questions for depression. Each item is measured on a four-point scale, with a score of 0 indicating “not present” and 3 indicating a “considerable event,” yielding a total score ranging from 0 to 21 on each of these two sub-scales. The cut-off score was 8 or more. A score greater than eight was considered indicative of an abnormal case for both the depression and anxiety subscales. For anxiety, the HADS has a specificity of 0.78 and a sensitivity of 0.9, and for depression it has a specificity of 0.79 and a sensitivity of 0.83 [12,21]. We used the Arabic version. Cronbach’s α was 0.83 (95% confidence interval: 0.79–0.88) for the HADS anxiety subscale and 0.77 (0.7–0.83) for the HADS depression subscale [21].

The questionnaire was reviewed by three experts in respiratory therapy field, and a pilot study was conducted with three patients to test the visibility, filling time, and applicability of the tool. The researchers assessed each CRD participant according to the inclusion criteria that mentioned above and explained the aim and objectives of the study. Then, participants who were willing to participate were asked to sign the informed consent and fill out the questionnaire. Each participant took 10–15 min to complete the questionnaire.

### 2.4. Ethical Considerations

The research proposal was approved by the Research and Ethical Committee at King Fahad Hospital Hofuf, Al Ahsa, Kingdom of Saudi Arabia (IRB KFHH NO. H-05-HS-065). Informed consent was obtained from the patients after explaining the aim of the study. Participation in this study was completely voluntary. The ability to withdraw from the study at any time and the confidentiality, privacy, and anonymity of the patients and their responses were assured. The study was conducted in accordance with the Declaration of Helsinki and followed ethical principles.

### 2.5. Statistical Analysis

Data were entered and analyzed using SPSS package version 28 (IBM, Armonk, NY, USA). Descriptive statistics were performed, and the results are presented as number (%) or mean (SD) for categorical and continuous variables, respectively. The normality of the data was graphically assessed. Correlation of PSQI with depression and anxiety was determined using Pearson’s correlation coefficient. Multiple linear regression analyses were performed to assess which patients’ characteristics were a significant predictor of poor sleep quality, depression, and anxiety. A *p* < 0.05 was considered significant, with a confidence interval of 95%. We also conducted multivariate liner regression to adjust for all the variables.

## 3. Results

There were 390 patients included in the current analysis. The mean (SD) age of the study population was 41.51 ± 11.85 years, and there were more females (58.5%) than males. The majority of the study participants were non-smokers and had no history of previous hospital admission. Patients with COPD and asthma were equally proportional. Baseline characteristics for the study participants are presented in Table 1.

### 3.1. Prevalence of Sleep Quality, Depression and Anxiety among Patients with CRDs

Table 2 shows the distribution of the PSQI components. The prevalence of poor sleep quality was 75% among all the CRDs patients. Of the total participant, a small proportion reported having very bad sleep (3.8%). The majority of the study population had a sleep latency of <60 min, and 65% of the study participants reported sleep disturbance at least once a week. In addition, more than half of the study participants reported having daytime dysfunction, and only a few patients were on sleep medications.

The prevalence of depression and anxiety among the patients living with CRDs was 49.2% and 36.4%, respectively (Table 3).

### 3.2. Correlation between Global PSQI Score, Total Anxiety, and Total Depression

There were positive correlations between the global PSQI with anxiety (r = 0.30, *p* < 0.001) and depression (r = 0.16, *p* < 0.001). In addition, anxiety and depression were positively correlated (r = 0.447, *p* < 0.001).

### 3.3. Predictors of Poor Sleep Quality among Patients with CRDs

A univariate linear regression model was performed to assess the predictors for poor sleep quality among patients with CRDs. The results showed that age, gender (female), family history of CRDs, smokers, dyspnea, depression, and anxiety were independently associated with worse sleep quality. In the multivariate regression model, age, gender, family history of CRDs and anxiety were significant predictors for PSQI, and these variables accounted for 0.18% of the variance in PSQI, as shown in Table 4.

### 3.4. Predictors of Depression and Anxiety among Patients with CRDs

The result of the association of depression (as the dependent variable) with patients’ characteristics and clinical data is presented in Table 5. Variables that independently predicted an increased level of depression were age, gender, marital status, family history of CRDs, diagnosis, the presence of comorbidities, dyspnea last month and anxiety. On the other hand, the variables that independently predicted an increased level of anxiety were age, BMI, family history of CRDs, previous hospital admission the presence of comorbidities, dyspnea last month and depression.

## 4. Discussion

To the best of our knowledge, this is the largest prospective study conducted in Al-Ahsa, Saudi Arabia, which investigated the prevalence of poor sleep quality, depression, and anxiety among patients with CRDs. Poor sleep quality, depression, and anxiety were highly prevalent in this group of patients, and there were multiple factors associated with the increased prevalence. Therefore, healthcare professionals should be vigilant to these conditions and target accordingly.

Although a large body of literature has investigated the prevalence of poor sleep quality in patients with CRDs, there is nevertheless a limited number of studies on the prevalence among the Saudi population. We found that 75% of patients included in our study had poor sleep quality (as measured by PSQI), slightly lower than the prevalence reported in a previous study (77% of 180) [22]. Methodological differences such as sample size and study design are likely to attribute to the difference in the reported prevalence. Importantly, current evidence suggests that poor sleep quality, which is highly prevalent in our cohort, has been associated with poor quality of life and poor survival in patients with COPD [23]. With the serious consequences associated with poor sleep quality, it is therefore of great importance to identify poor sleep quality among this population, and intervene accordingly.

Mechanisms linking poor sleep quality to patients with CRDs remain to be ascertained. However, there have been a number of factors suggested. Previous data from the Tucson Epidemiologic Study of Chronic Lung Disease showed a strong relationship between respiratory symptoms and poor sleep quality [24]. Symptoms, such as cough and wheezing, which are almost always presented in patients with CRDs, have been associated with increased rates of insomnia and daytime sleepiness in comparison to subjects without respiratory symptoms. In addition, dyspnea, which is the hallmark of COPD, accumulation of secretion with associated mucus plugging, and worse ventilation and oxygenation have also shown to play a major role in affecting sleep quality [25,26].

Sleep quality is important for the psychological and physical health of chronic respiratory patients [27]. The current study found that the highest component score of sleep quality was sleep latency. When asked how long it took them to fall asleep, 32.8% of the participants answered “16–30 min,” while 32.1% answered “31–60 min.” A retrospective study conducted in 2016 [28] seeking to identify the factors associated with sleep disturbance in patients with COPD found that the PSQI component with the highest score was “getting up to use the bathroom” (70.3%).

Findings of the current study reveal that sleep efficiency is present in more than half (58.2%) of the study population. This can be attributed to the fact that the majority of the participants reported not using sleep medications during the past month and having a sleep disturbance “Less than once a week.” This was in line with a prior study [29] in which more than half (53% of 1117) of the participants with COPD reported “poor” sleep quality. On the contrary, a study [30] reported that the incidence of poor sleep quality was only 35% (N = 245) in patients with CRDs.

Anxiety and depression are common psychological disturbances in patients with CRDs, with a significant effect on health and prognosis. A randomized cross-sectional study [31] reported that 12% of 280 patients were diagnosed with depression among the screened participants. They presented to primary health care centers in Sharurah Armed Forces Hospital (SAFH), Sharurah, Saudi Arabia. In the present study, we found that the prevalence of depression and anxiety to be 49.2% and 36.4%, respectively. This is in-line with the previous literature, which estimates that 10–57% of patients with CRDs have anxiety and 10–59% have depression [32,33]. In general, patients with CRDS, such as COPD, have increased risk of anxiety and depression compared to controls or to patients with other chronic diseases [34,35]. Current evidence also shows that the prevalence increases along with the severity of respiratory symptoms. Here, we report that dyspnea was a significant predictor of anxiety and depression. In a large population-based study, Siraj et al. [36] showed COPD patients with severe respiratory symptoms (dyspnea) compared to patients with less severe symptoms. As dyspnea is a modifiable factor, and has been independently linked to increased anxiety and depression in patients with CRDs, it is therefore important to consider approaches which alleviate dyspnea, one of which is pulmonary rehabilitation.

Several factors contribute to the increases in prevalence of mental health, anxiety and depression, in patients with CRDs: age, gender and severity of the disease. In this study, the female gender was associated with an increased prevalence of anxiety and depression. A previous study [37] demonstrated that female patients with COPD were at increased risk of depression compared to male patients. This is also true among subjects with CRDs. The reasons for increased risk of mental health among females—regardless of the presence of CRDs—are brain structure, function, stress and hormones. However, this does not mean to underestimate the burden of mental health among male patients, as anxiety and depression remain a major issue for both [38].

The findings that anxiety and depression are directly related in our study are concordant with prior literature. Indeed, previous studies estimates suggest that 26–43% of patients with CRDs, mainly COPD, have both anxiety and depression. In addition, studies [39,40] also show that COPD patients with depression are more likely to develop anxiety compared to COPD patients without depression. It is worth noting, however, that each condition alone has a significant impact on patients’ health. When both conditions co-exist in patients with CRDs, the impact is likely to be magnified. Indeed, there is evidence to suggest that patients with anxiety and depression are at greater risk of suicidal thoughts and physical disability compared to patients with either condition. Thus, routine assessment mental health in patients with CRDs should be prioritized.

The current study found a strong positive correlation between the PSQI sleep quality score and both HDAS depression and anxiety scores. This is consistent with a previous study that found a relationship between sleep disturbance and depression [41]. Moreover, similar to our findings, the earlier studies found association between sleep disturbances and both depression and anxiety scores in patients with COPD [37].

The main strengths of current study are its large sample size (based on a sample calculation) and the inclusion of patients with confirmed diagnosis (either COPD or Asthma) with co-existing comorbidities, a different approach that has not been widely used in previous studies. It is therefore representative to the typical profile of patients with CRDs seen at the clinical settings. However, this study has some limitations. First, the cross-sectional nature of the study did not allow assessment for any causality. Second, it was not possible to use the pulmonary function test (PFT) to confirm the diagnosis and the severity of the disease, due to the infection control precautions imposed by the Ministry of Health due to COVID-19. However, all participants have been recently diagnosed with CRDs by their physicians. Lastly, we have no information on occupational status, which would add more data to future studies. This work highlights the need for continuous screening for sleep quality, depression, and anxiety among patients with CRDs, and it recommends the development of interventions and/or management protocols to help to improve their quality of life.

## 5. Conclusions

There is a high prevalence of poor sleep quality among patients with CRDs, and it is significantly correlated with depression and anxiety. The predictors of poor sleep quality were age, gender, family history of CRDs education level and anxiety. Variables that independently predicted an increased level of depression were age, gender, marital status, family history of CRD, diagnosis, previous admissions, the presence of comorbidities, dyspnea last month and anxiety. The variables that independently predicted an increased level of anxiety were age, BMI, family history of CRDs, previous hospital admission, the presence of comorbidities, dyspnea last month and depression. We recommend that future studies should adopt the PFT and consider objective assessments of sleep quality.

## Figures and Tables

**Table 1:** Distribution of characteristics of patients with chronic respiratory diseases (N = 390).

Characteristics	N (%) Mean SD	
**Age**	41.51 ± 11.85	
**Gender**		
Male	162	41.5
Female	228	58.5
**Marital status**		
Single	165	42.3
Married	225	57.7
**Education level**		
Pre-university	284	72.8
University level	106	27.2
**BMI**		
Underweight	4	1.0
Normal weight	105	26.9
Overweight	179	45.9
Obese	102	26.2
**Family history of respiratory diseases**		
Yes	48	12.3
No	342	87.7
**Smoking (lifetime)**		
Yes	165	42.3
No	225	57.7
Diagnosis		
COPD	190	48.7
Asthma	200	51.3
**Previous hospital admission for exacerbation**		
Yes	63	16.2
No	327	83.8
**Comorbid Illness**		
None	294	75.4
Anemia	23	5.9
Hypertension	46	11.8
Diabetes Mellitus	24	6.2
Dysrhythmia	3	0.8

**Table 2:** Distribution of sleep quality assessment (PSQI) of patients with chronic respiratory disorders (N = 390).

Sleep Quality Assessment (PSQI)	
Component Scores	N (%)
**Subjective sleep quality**	
▪ Very good	100 (25.6)
▪ Fairly good	233 (59.7)
▪ Fairly bad	42 (10.8)
▪ Very bad	15 (3.8)
**Sleep latency**	
▪ <15 min	46 (11.8)
▪ 16–30 min	128 (32.8)
▪ 31–60 min	125 (32.1)
▪ >60 min	91 (23.3)
**Sleep duration**	
▪ >7 h	97 (24.9)
▪ 6–7 h	113 (29.0)
▪ 5–6 h	92 (23.6)
▪ <5 h	88 (22.6)
**Sleep efficiency**	
▪ >85%	227 (58.2)
▪ 75–84%	87 (22.3)
▪ 65–74%	38 (9.7)
▪ <65%	38 (9.7)
**Sleep disturbance**	
▪ Not in the past month	0 (0)
▪ Less than once a week	254 (65.1)
▪ Once or twice a week	136 (34.9)
▪ Three or more times a week	0 (0)
**Use of sleep medication**	
▪ Not in the past month	297 (76.2)
▪ Less than once a week	52 (13.3)
▪ Once or twice a week	34 (8.7)
▪ Three or more times a week	7 (1.8)
**Daytime dysfunction**	
▪ Not in the past month	164 (42.1)
▪ Less than once a week	105 (26.9)
▪ Once or twice a week	105 (26.9)
▪ Three or more times a week	16 (4.1)
**Global PSQI**	
▪ Good Sleep Quality (≤5)	97(24.9)
▪ Poor Sleep Quality(≥5)	293(75.1)

**Table 3:** Distribution of total scores of Hospital Anxiety and Depression among patients with CRD N = 390.

Variables	N (%)
**Depression (Total Score)**	
▪ Normal (0–7)	198 (50.8)
▪ **Abnormal (8–21)**	192 (49.2)
**Anxiety (Total Score)**	
▪ **Normal (0–7)**	248 (63.6)
▪ **Abnormal (8–21)**	142 (36.4)

**Table 4:** Regression analyses of poor sleep quality based on the characteristics of patients with chronic respiratory diseases (N = 390).

Independent Variables	Model 1: β (95% CI)	*p* Value	Model 2: β (95% CI)	*p* Value
**Age**	−0.035 (−0.061, −0.009)	0.008	−0.036 (−0.066, −0.007)	0.016
**Gender** **(Male)**	1.338 (0.721, 1.956)	0.001	1.244 (0.488, 2.000)	0.001
**Marital status** **(Married)**	0.566 (−0.062, 1.194)	0.077	0.358 (−0.261, 0.976)	0.256
**BMI**	−0.006 (−0.083, 0.072)	0.884	−0.014 (−0.042, 0.013)	0.302
**Family history of CRDs** **(No)**	−1.649 (−2.582, −0.716)	0.001	−1.789(−2.720, −0.858)	<0.001
**Smoking** **(lifetime)** **(Yes)**	0.965 (0.342, 1.588)	0.002	0.291 (−0.540, 1.121)	0.492
**Education level** **(Pre-university level)**	−0.916 (−1.607, −0.224)	0.010	−1.009 (−1.704, −0.314)	0.005
**Diagnosis** **(Yes)**	0.727 (0.111, 1.342)	0.021	−0.660 (−1.494, 0.175)	0.121
**Previous hospital admission** **(No)**	−0.542 (−1.386, 0.303)	0.208	−0.033 (−0.932, 0.866)	0.942
**Comorbid illness** **(Yes)**	0.076 (−0.244, 0.395)	0.642	0.247 (−0.077, 0.571)	0.135
**Dyspnea last month** **(Yes)**	1.117 (−1.729, −0.504)	0.001	0.028 (−0.653, 0.709)	0.936
**Depression** **(Yes)**	0.218 (0.087, 0.348)	0.001	0.084 (−0.003, 0.171)	0.058
**Anxiety** **(Yes)**	0.354 (3.245, 5.040)	0.001	0.177 (0.084, 0.270)	<0.001

**Model 1**: Univariate association of PSQI and other variables. **Model 2**: Multivariable associations of PSQI as the dependent variable and age, gender, marital status, BMI, family history of CRDs, smoking, education level, diagnosis, previous hospital admission, comorbid illness, dyspnea last month, depression and anxiety as independent variables (Model R square = 0.19, *p* value < 0.001).

**Table 5:** Regression analysis of depression and anxiety scores based on the characteristics of patients with chronic respiratory diseases (N = 390).

Independent Variables	Model 1: β (95% CI)	*p* Value	Model 2: β (95% CI)	*p* Value
**Depression score**				
**Age**	0.008 (−0.027, 0.043)	0.670	0.043 (0.009, 0.077)	0.014
**Gender** **(Male)**	−1.202 (−2.034, −0.371)	0.005	−2.129 (−2.983, −1.275)	<0.001
**Marital status** **(Married)**	1.223 (0.394, 2.051)	0.004	1.005 (0.291, 1.719)	0.006
**BMI**	0.028 (−0.067, 0.138)	0.110	−0.012 (−0.044, 0.020)	0.454
**Family history of CRDs** **(Yes)**	0.036 (−0.803, 1.715)	0.498	1.666 (0.594, 2.738)	0.002
**Smoking** **(lifetime)** **(Yes)**	0.122 (−0.960, 0.716)	0.775	0.093 (−0.876, 1.061)	0.851
**Education level** **(Pre-university level)**	−098 (−1.026, 0.831)	0.836	−0.059 (−0.870, 0.751)	0.885
**Diagnosis** **(Yes)**	0.956 (0.130, 1.783)	0.023	1.361 (0.398, 2.324)	0.006
**Previous hospital admission** **(Yes)**	0.457 (−4.672, −2.541)	0.098	−0.272 (−4.040, −2.096)	<0.001
**Comorbid illness** **(No)**	−3.607 (−0.705, 0.144)	0.001	−2.751 (−3.762, −1.740)	<0.001
**Dyspnea last month** **(No)**	−0.281 (−3.583, −2.025)	0.194	−1.452 (−2.232, −0.672)	<0.001
**Anxiety** **(Yes)**	0.506 (0.405, 0.607)	<0.001	0.394 (0.293, 0.496)	<0.001
**Anxiety Score**				
**Age**	−0.037 (−0.067, −0.006)	0.020	−0.041 (−0.073, −0.010)	0.011
**Gender** **(Male)**	−0.005 (−0.748, 0.738)	0.990	−0.442 (−1.261, 0.377)	0.289
**Marital status** **(Married)**	0.471 (−0.269, 1.210)	0.212	−0.430 (−1.100, 0.240)	0.208
**BMI**	0.059 (−0.032, 0.150)	0.203	−0.041 (−0.071, −0.012)	0.006
**Family history of CRDs** **(No)**	−0.848 (−1.959, 0.263)	0.134	−1.177 (−2.180, −0.173)	0.022
**Smoking** **(lifetime)** **(Yes)**	0.397 (−0.343, 1.137)	0.292	0.023 (−0.925, 0.878)	0.959
**Education level** **(Pre-university level)**	−0.713 (−1.529, 0.103)	0.086	−0.747 (−1.498, 0.003)	0.051
**Diagnosis** **(Yes)**	0.970 (0.244, 1.696)	0.009	0.462 (−0.443, 1.366)	0.316
**Previous hospital admission** **(No)**	−1.048 (−2.037, −0.059)	0.038	1.055 (0.085, 2.025)	0.033
**Comorbid illness** **(Yes)**	0.088 (−0.288, 0.464)	0.646	0.445 (0.096, 0.794)	0.013
**Dyspnea last month** **(No)**	−2.659 (−3.341, −1.977)	<0.001	−1.967 (−2.679, −1.256)	<0.001
**Depression** **(Yes)**	0.506 (0.405, 0.607)	<0.001	0.342 (0.254, 0.429)	<0.001

**Model 1:** Univariate association of depression and anxiety scores and other variables. **Model 2:** Multivariable associations of depression and anxiety scores as the dependent variable and age, gender, marital status, BMI, family history of CRDs, smoking, education level, diagnosis, previous hospital admission, comorbid illness, dyspnea last month, depression and anxiety as independent variables (Model R square for depression = 0.38.2, *p* value < 0.001, Model R square for anxiety = 0.316, *p* value < 0.001).

## Data Availability

The data presented in this study are available on reasonable request from the corresponding author.
